# Longitudinal changes in the hypothalamic–pituitary–adrenal axis and sympathetic nervous system are related to the prognosis of stroke

**DOI:** 10.3389/fneur.2022.946593

**Published:** 2022-07-27

**Authors:** Xu-Guang Chen, Sheng-Yi Shi, Lan Hu, Yu Chen, Han-Wen Sun, Lei Zhou, Zhen-Bing Lu, Huan Wang, Xiao-Shan Wang, Jie Yu, Yu-Jia Zhao, Yi-Ming Lu, Jing Ye

**Affiliations:** ^1^Department of Geriatrics, Medical Center on Aging of Shanghai Ruijin Hospital, Shanghai Jiaotong University School of Medicine, Shanghai, China; ^2^Emergency Department of Shanghai Ruijin Hospital, Shanghai Jiaotong University School of Medicine, Shanghai, China; ^3^Shanghai Nanxiang Hospital Affiliated to Shanghai Ruijin Hospital, Shanghai, China

**Keywords:** stroke, hormones, catecholamines, sympathetic, severity, prognosis

## Abstract

**Background and purpose:**

This study sought to improve methods to identify biomarkers in the neuroendocrine system related to stroke progression to improve the accuracy of traditional tools for evaluating stroke prognosis.

**Methods:**

Seventy-four stroke patients and 237 healthy controls were prospectively included. We measured urinary epinephrine (E), noradrenaline (NE), dopamine (DA) and cortisol (F) on days 1, 3, and 5 after stroke onset and plasma F, adrenocorticotropic hormone (ACTH), thyrotropin (TSH), prolactin (PRL), follicle-stimulating hormone (FSH), luteinizing hormone (LH) and growth hormone (GH). The correlation between these hormone levels and 90-day prognosis was analyzed, their value in assessing prognosis was compared with lesion volume and National Institutes of Health Stroke Scale (NIHSS) scores using receiver operating characteristic (ROC) curves, and their correlation with conventional clinical variables was assessed.

**Results:**

Levels of F, 24-h urinary free cortisol(UFC), E, NE, DA, and GH on days 1, 3, and 5 were significantly higher in stroke patients than in controls (*P* < 0.01), while ACTH and TSH decreased, gradually approaching normal within 5 days of onset. Levels of E, NE, F, and 24-h UFC were proportional to severity, and all gradually decreased within 5 days of onset in patients with a good prognosis and gradually increased or remained high in those with a poor prognosis. After adjustment for age, sex, NIHSS, or Glasgow Coma Scale (GCS) score, *F* > 13.6 μg/dL, ACTH > 22.02 pg/mL and NE > 123.5 μg/ 24 h were identified as risk factors for a poor prognosis 90 days after stroke (*P* < 0.05). The combination of F, ACTH, NE, white blood cell count (WBC), glucose (Glu), and hemoglobin (Hb) was significantly more accurate than lesion volume (AUC: 0.931 vs. 0.694 *P* = 0.019) and NIHSS score (AUC: 0.931 vs. 0.746 *P* = 0.034) in predicting poor prognosis of stroke 1 day after onset. Hormones and traditional clinical variables were correlated to varying degrees, with NE correlating most strongly with 24-h UFC (*r* = 0.54) and moderately positively with lesion volume (*r* = 0.40) and NIHSS score (*r* = 0.45).

**Conclusions:**

Stroke causes significant time-phased dynamic changes in the hypothalamic–pituitary–adrenal axis and sympathetic nervous system, and plasma F, ACTH, and urinary NE levels can be used to assess stroke severity and prognosis.

**Chinese clinical trial registry:**

Registration Number: ChiCTR1900024992. Registration Date: 2019/8/6.

Stroke is a leading cause of death worldwide (1), with 6.2 million people dying from stroke in 2017, which is nearly two times greater than the number of deaths in 1990 (2); approximately 3 to 4% of health care expenditures in Western countries are spent on stroke (3). Reducing stroke-related mortality to decrease the socioeconomic burden is a global challenge.

Early and accurate assessment of stroke severity and early risk and prognostic analysis are essential for making the right clinical decisions to improve prognosis. The National Institutes of Health Stroke Scale (NIHSS) is commonly used internationally as a standardized and widely used assessment for predicting the 3-month prognosis of acute cerebrovascular events. However, its use requires specific training, and there is observer subjectivity. Other scale assessment methods, such as the European Stroke Scale (ESS), the Canadian Neurological Scale (CNS) and the Scandinavian Stroke Scale (SSS), for assessing the prognosis of stroke have the same limitations. The scale method suffers from interrater inconsistency (4) and underestimates the risk of posterior circulation stroke (5). Therefore, there is a clinical need for simple and objective assessment tools for predicting disease progression, outcome and mortality.

Neuroimaging has made great advances in recent years and provides a more objective and quantitative assessment for understanding the severity and prognosis of stroke. In addition to imaging, biomarker studies related to stroke prognosis are known as hot spots; however, a systematic evaluation found that although these studies involve neuroendocrine aspects, not many (6, 7) involve the detection of central endocrine metabolites, which can be useful in the assessment of stroke. The classic 'stress response' of the body to stroke occurs with hypothalamic–pituitary–adrenal (HPA) stressors. In cerebral ischemia, HPA changes are one of the first measurable endocrine changes and are characterized by increased cortisol levels, decreased thyroid function and deficits in the synthesis of metabolic hormones such as growth hormone (GH) and insulin. Other anterior pituitary axis hormones, such as peripheral thyroid hormone and growth hormone, are also useful in predicting stroke prognosis (8), which is associated with immune dysregulation due to neuroendocrine disruption after stroke. Low T3 syndrome is an independent predictor of survival among acute stroke patients, and even low T3 levels within the normal range have been associated with a poorer prognosis among acute stroke patients (9). Patients with higher levels of growth hormone have a higher mortality rate and a poorer prognosis, which are associated with stress-mediated increases in GH levels (10).

Although the prevalence of neuroendocrine system changes during the acute phase of stroke is being increasingly recognized, the dynamics of key neuroendocrine factors of the organism, other than cortisol, GH and T3, in stroke onset and their relationship with prognosis have been less well studied (8, 11–13). Although some studies have found potential mechanisms for the presence of brain–heart interactions in stroke related to the HPA axis. For example, catecholamines may mediate neurogenic heart damage after brain injury (14), and the HPA axis is associated with poststroke infection (15). However, there is a lack of research on whether these mechanisms are related to each other. In addition, HPA axis modification therapy for stroke has been unsuccessful in translation because of the poor agreement between the therapeutic window of corticotropin-releasing factor antagonism and the pharmacokinetics of the explored antagonists (16). Studies of central noradrenergic agonists for stroke have encountered similar problems (17). Therefore, a comprehensive understanding of endocrine changes in different periods after stroke is essential for the development of new drug targets.

In conclusion, although the central endocrine metabolic pathway reflects, to some extent, the neurotransmitter regulation of brain tissue and the performance of this function is closely related to the number and function of central neuronal cells, the complexity of the regulation and interaction of the central endocrine metabolic pathway, especially the rhythmic nature of endocrine and the variability during different stages of disease, greatly limit the use of endocrine-related biomarkers. We designed a prospective cohort study to investigate the dynamic expression of these central endocrine metabolites in the plasma of stroke patients under the premise of strictly controlling the endocrine rhythm. In addition, in order to avoid the issue of large fluctuations in plasma catecholamine levels, we selected urine samples with more stable catecholamine levels. We measured serum adrenocorticotropic hormone (ACTH), plasma cortisol (F), 24-h urinary free cortisol (UFC), luteinizing hormone (LH), and urine cortisol on days 1, 3, and 5 after stroke onset. LH, follicle-stimulating hormone (FSH), prolactin (PRL), urinary free epinephrine (E), norepinephrine (NE), dopamine (DA), thyroid-stimulating hormone (TSH), and GH were also quantified. This study aimed to analyze the correlation of these central endocrine metabolites in the early stage of stroke with prognosis at 90 days, to explore their roles in aiding the more commonly used or recognized current clinical tools for stroke severity and prognosis assessment, such as NIHSS scores, Glasgow Coma Scale (GCS) scores and imaging lesion volumes, and to investigate the value of these central endocrine metabolites for prognostic assessment.

## Methods

### Study population

Patients with stroke who visited the emergency department of the Ruijin Hospital North Campus, Shanghai Jiaotong University School of Medicine, from May 2019 to January 2021 were prospectively selected. The inclusion criteria were as follows: (1) A diagnosis of stroke in accordance with the 2013 update of the American Heart Association/American Stroke Association definition of stroke (18); (2) arrival within 24 h of stroke onset; (3) age >18 years; (4) first onset; (5) GCS score >8; and (6) provision of signed informed consent. The exclusion criteria were (1) the presence of complications of epilepsy, infection, or gastrointestinal bleeding; (2) the use of vasoactive and sympathomimetic active drugs within 1 week before and after admission; (3) exclusion of subarachnoid hemorrhage and intraventricular hemorrhage, taking into account differences in prognostic assessment; (4) a history of psychiatric disease, thyroid disease, pituitary insufficiency, renal disease, severe cardiac insufficiency, respiratory failure, hepatic insufficiency, or malignancy; and (5) surgical treatment. Patients received standard treatment in the emergency stroke unit for hemorrhagic stroke (HS) and ischemic stroke (IS) in accordance with the American Heart Association/American Stroke Association recommended guidelines for the treatment of spontaneous cerebral hemorrhage (2015) (19) and with the American Heart Association/American Stroke Association recommended guidelines for the early treatment of patients with acute ischemic stroke 2018, respectively (20). Individuals who underwent health check-ups at our center during the same period were selected as healthy controls. All patients or family members signed a written informed consent form.

### Baseline data acquisition

All patients were evaluated by 2 designated emergency department physicians. The demographic and clinical characteristics included age, sex, body mass index, stroke type, lesion site, GCS score, NIHSS score, vital signs on admission, past medical history, preadmission medication history and the presence of hypertension (HTN), diabetes mellitus (DM), hyperlipidemia (HL), coronary artery disease (CAD), and smoking-related vascular risk factors. The GCS assesses the state of consciousness by eye opening response, speech, and movement. The NIHSS is used to assess the degree of functional impairment caused by stroke and consists of a total of 11 tests with a score range of 0 to 42, with higher scores indicating more severe stroke. Routine hematological investigations, including routine blood tests, hepatic and renal function, coagulation, fasting glucose and lipids, were completed within 24 h of admission and cerebrovascular and cervical angiography within 72 h of admission. CT and diffusion-weighted imaging (DWI) lesion volumes were assessed using MIPAV software (version 11.0) by two experienced radiologists with no knowledge of the clinical and laboratory findings.

### Specimen test

Collection of 24-h urine was started immediately after admission. The urine collection bottle was prefilled with 5% glacial acetic acid to decrease the urine pH to <4.0, the specimen was protected from light, and the 24-h urine volume was recorded. Urine specimens were sent to the Shanghai Institute of Hypertension at 8:00 a.m. on days 1, 3, and 5 after admission for assessment of urinary free E, NE, and DA levels within 2 h by high-performance liquid chromatography (instrument provided by Agilent Technologies, Ltd., with the corresponding reagents); 5 ml of peripheral cubital venous blood was collected from the subjects at 8:00 after an overnight fast on days 1, 3, and 5 after admission and sent to the hospital laboratory within 30 min of collection. Twenty-four-hour UFC, F, PRL, FSH and LH levels were measured by chemiluminescence (instrument: Beckman DXI800, reagents in kit), TSH levels were measured by electrochemiluminescence (instrument: Abbott i2000, reagents in kit), and GH and ACTH levels were measured by electrochemiluminescence (instrument: Roche Cobas 601, reagents in kit).

### Outcomes

The event endpoint was functional outcome 90 days after onset. The modified Rankin Scale (mRS) score was obtained by a standardized telephone interview with the patient or a family member by 1 trained doctor 90 days after onset, with a good outcome defined as an mRS score ≤ 2. Briefly, a Rankin Scale score of 0 indicates no symptoms; a score of 1 indicates no evident disability despite symptoms; a score of 2 indicates slight disability, with an inability to carry out all previous activities; a score of 3 indicates moderate disability, with the need for some help but the ability to walk without assistance; a score of 4 indicates moderately severe disability, with the inability to walk without assistance or to attend to bodily needs without assistance; a score of 5 indicates severe disability, with the patient being bedridden and incontinent and requiring constant nursing care; and a score of 6 indicates death.

### Grouping

Patients were divided into groups according to the type of stroke (hemorrhagic stroke and ischemic stroke), the NIHSS score [a light group (<10 points) and a heavy group (≥10 points)], and the mRS score [a good prognosis group (mRS ≤ 2 points) and a poor prognosis group (mRS >2 points)].

### Statistical analysis

Data were statistically analyzed using SPSS 23.0 and MedCalc software. Categorical variables are expressed as frequencies (component ratios) and were compared using the chi-square test. Non-normally distributed variables are presented as the median (interquartile range). The Mann–Whitney *U* test and Kruskal–Wallis test were used to compare two or more sample, and Spearman's correlation was calculated between different variables. Univariate regression models were used to assess the accuracy of biomarkers and other clinical variables in predicting prognosis. We did not perform multivariate analyses due to the limited number of results and the risk of overfitting. A *P* value < 0.05 indicated a statistically significant difference, and the results were plotted using GraphPad Prism software (version 8.0.2).

## Results

### Patients

A total of 74 stroke patients were enrolled, excluding 2 cases of postadmission sedation, 3 cases of concurrent infection, 1 case of concurrent gout attack, 1 case of moyamoya disease found after admission and 1 case of postdischarge death due to a traffic accident. Sixty-six patients were included in the analysis (Supplementary Figure S1), comprising 29 patients with hemorrhagic stroke [median age, 55 years (IQR 49–64); 21 males and 8 females] and 37 patients with ischemic stroke [median age, 67 years (IQR 52–74); 22 males and 15 females]. The prognosis was good in 40 cases and poor in 26 cases at the 90-day follow-up, with most patients having one or two risk factors, most commonly hypertension. In the same period, 237 healthy individuals were included in the healthy control group. There was no significant difference in age or sex between the stroke patient and healthy control groups (Table 1).

**Table 1:** Analysis of baseline characteristics of the stroke patient and healthy control groups.

	**Stroke patients**	**Healthy controls**	***P*** **-value**
	**(*****N*** = **66)**	**(*****N*** = **237)**	
Age, *y*	61.1 (± 13.1)	61.0 (± 9.5)	0.980
Male	43 (65.2%)	137 (57.8%)	0.282

*Data are presented as means ± SDs for continuous variables and as n (%) for categorical variables*.

*N indicates the total number of cases in different groups*.

### Changes in hormone levels at various time points

**Figure 1:**
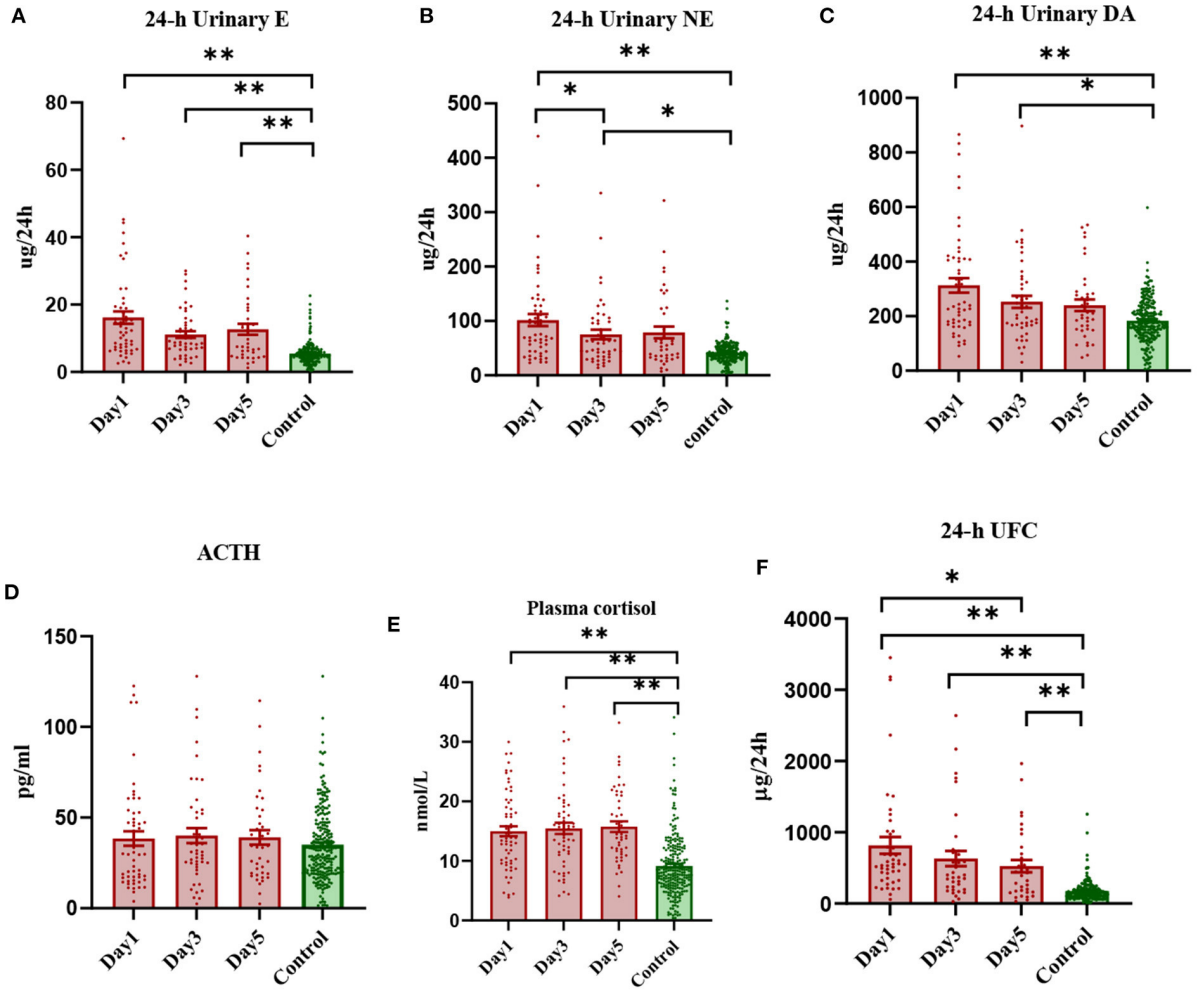
Scatterplot of early levels of each hormone in stroke patients vs. healthy controls (median and interquartile range). *Denotes *P* < 0.05, **denotes *P* < 0.01.

Compared with those in the healthy control population, the levels of 24-h urinary E, NE and DA in stroke patients increased significantly, and gradually decreased with the passage of time. Among them, the levels of 24-h NE and DA were similar to those in the healthy group [IQR 53.98 (33.23, 120.5) ug/24 h vs. 40.05 (31.43, 52.22) ug/24 h], but the level of 24-h E was still significantly higher than that in the healthy group on the 5^th^ day after onset (Figures 1A–C). F and 24-h UFC levels in the HPA axis pathway were significantly higher in stroke patients, with F levels remaining high for 5 days and 24-h UFC levels gradually decreasing over 5 days, but still significantly higher than those in the healthy control population (Figures 1E,F). ACTH levels decreased slightly on the first day after stroke compared to those in the healthy control group [IQR 29.74 (16.55, 52.54) pg/ml vs. 32.07 (20.10, 46.07) pg/ml], and then gradually increased slightly [IQR 29.74 (16.55, 52.54) pg/ml; 31.08 (24.14, 52.94) pg/ml; 34.06 (19.11, 53.28) pg/ml], but the difference was not statistically significant (Figure 1D). The trend in anterior pituitary hormone levels was inconsistent in the stroke patient population compared to the healthy control population, with GH levels increasing significantly after onset [IQR 0.356 (0.161, 0.738) μg/L vs. 0.137 (0.057, 0.411) μg/L] and remaining high for 5 days, TSH levels decreasing significantly [IQR 0.7833 (0.4215, 1.2340) mIU/L vs. 1.9290 (1.2080, 2.8040) mIU/L] and then increasing gradually, and FSH, LH and PRL levels showing no significant change (Supplementary Figure S2).

### Baseline characterization

Among the main baseline characteristics of the case group at admission (Table 2), chi-square test and Mann–Whitney *U* test analyses showed that compared with the good prognosis group, the poor prognosis group had higher NIHSS scores (12 vs. 6), lower GCS scores (13 vs. 15), and larger lesion volume (15 cm^2^ vs. 4.4 cm^2^), WBC was higher (9.03 × 10^9^·L^−1^ vs. 7.21 × 10^9^·L^−1^), blood glucose was higher (8.9 mmol·L^−1^ vs. 6.3 mmol·L^−1^), hemoglobin level was lower (126 g·L^−1^ vs. 139 g·L^−1^), 24-h NE level was higher (116.71 μg·24 h^−1^ vs. 68.25 μg·24 h^−1^), ACTH level was lower (20.20 pg·ml^−1^ vs. 42.72 pg·ml^−1^), FSH level was higher (36.40 mIU·mL^−1^ vs. 8.71 mIU·mL^−1^), and blood cortisol level was higher (15.66 μg·dL^−1^ vs. 12.82 μg·dL^−1^). There were no significant differences in stroke type, heart rate, blood pressure, site of lesion, risk factors, liver and kidney function, lipids or coagulation, E, DA, LH, PRL, TSH, GH, or 24-h UFC between the prognosis groups.

**Table 2:** Baseline characteristics of stroke patients on admission to hospital.

	**Total (*****n*** = **66)**	**90-day mRS score** ≤ **2**	**90-day mRS score** > **2**	***P*** **-value**
Baseline characteristic				
NIHSS score, median (IQR)	10 (4.8–13.3)	6 (3.11)	12 (10.16)	0.000
GCS score, median (IQR)	15 (13–15)	15 (14.15)	13 (12.15)	0.000
BMI, median (IQR), kg·m^−2^	23.9 (22.0–26.3)	24.9 (22.2–27.0)	23.0 (21.5–25.0)	0.050
HR (beats·min^−1^)	80 (75–87)	80 (74–87)	82 (75–88)	0.373
SBP (mmHg)	149 (138–165)	150 (139–163)	149 (134–167)	0.971
DBP (mmHg)	85 (77–95)	87 (80–97)	80 (74–94)	0.218
Stroke type, *n* (%)				
HS	29 (43.9%)	16 (24.2%)	13 (19.7%)	0.424
IS	37 (56.1%)	24 (36.4%)	13 (19.7%)	
Stroke location, *n* (%)				
Basal ganglia	31 (47.0%)	20 (30.3%)	11 (16.7%)	0.803
Thalamus	8 (12.1%)	4 (6.1%)	4 (6.1%)	
Cerebellum	7 (10.6%)	5 (7.6%)	2 (3.0%)	
Occipital lobe	2 (3.0%)	1 (1.5%)	1 (1.5%)	
Frontal lobe	7 (10.6%)	3 (4.5%)	4 (6.1%)	
Parietal lobe	1 (1.5%)	0 (0.0%)	1 (1.5%)	
Temporal lobe	2 (3.0%)	1 (1.5%)	1 (1.5%)	
Cerebellum	1 (1.5%)	1 (1.5%)	0 (0.0%)	
Near tricorn	7 (10.6%)	5 (7.6%)	2 (3.0%)	
Risk factor, *n* (%)				
Hypertension	45 (68.2%)	25 (37.9%)	20 (30.3%)	0.219
Diabetes mellitus	19 (28.8%)	9 (13.6%)	10 (15.2%)	0.162
Hypercholesterolemia	28 (42.4%)	18 (27.2%)	10 (15.2%)	0.599
Smoking	21 (31.8%)	12 (18.2%)	9 (13.6%)	0.694
Imageological examination				
Lesion volume, median (IQR), cm^2^	6.6 (1.3–20.0)	4.4 (1.2–7.7)	15.0 (1.2–33.3)	0.026
Routine laboratory inspection				
White blood cells (10^9^·L^−1^)	8.09 (6.56–9.64)	7.21 (6.17–9.13)	9.03 (6.94–13.89)	0.015
Glucose (mmol·L^−1^)	6.9 (5.8–9.6)	6.3 (5.6–8.5)	8.9 (6.6–11.1)	0.004
Hemoglobin (g·L^−1^)	137 (125–145)	139 (131–149)	126 (118–139)	0.008
HbA1c (%)	5.9 (5.6–6.6)	5.9 (5.6–6.35)	5.95 (5.35–8.45)	0.925
LDL(mmoI·L^−1^)	3.06 (2.62–3.77)	3.16 (2.68–3.79)	3.00 (2.49–3.70)	0.399
Platelets (×10^9^·L^−1^)	176 (151–217)	167 (149–202)	191 (172–241)	0.111
Gamma-glutamyl transferase	19 (13–28)	19 (14–26)	21 (13–34)	0.280
(IU·L^−1^)				
Albumin (g·L^−1^)	39 (36–43)	39 (37–43)	38 (36–42)	0.609
Creatinine (μmoI·L^−1^)	76 (65–85)	76 (66–83)	73 (63–92)	0.813
INR	1.03 (0.97–1.08)	1.03 (0.98–1.07)	1.03 (0.97–1.12)	0.465
D-dimer (μg·mL^−1^)	0.14 (0.07–0.32)	0.12 (0.06–0.22)	0.20 (0.08–0.68)	0.060
CRP (mg·dl^−1^)	10.0 (10.0–10.8)	10.0 (10.0–10.0)	10.0 (10.0–13.8)	0.081
24-h urinary free catecholamines day 1				
Epinephrine (μg·24 h^−1^)	12.04 (6.89–19.32)	10.50 (6.68–18.64)	14.15 (7.47–23.31)	0.347
Norepinephrine (μg·24 h^−1^)	74.55 (44.24–128.25)	68.25 (42.21–109.31)	116.71 (65.23–177.26)	0.033
Dopamine (μg·24 h^−1^)	239.74 (172.60–411.27)	239.74 (183.87–413.33)	215.58 (160.57–398.10)	0.388
Anterior pituitary hormones day 1				
Adrenocorticotropic (pg·ml^−1^)	29.74 (16.55–52.54)	42.72 (18.64–60.51)	20.20 (13.62–46.62)	0.047
Luteinizing hormone (μIU·mL^−1^)	9.35 (3.93–19.50)	6.54 (3.44–15.87)	14.00 (5.12–26.13)	0.072
Follicle-stimulating hormone	14.20 (5.47–48.20)	8.71 (4.70–37.72)	36.40 (8.99–56.99)	0.015
(mIU·mL^−1^)				
Prolactin (ng·ml^−1^)	10.66 (7.58–15.01)	10.74 (7.29–14.03)	10.26 (8.33–15.18)	0.748
Thyroid-stimulating hormone	0.8102 (0.4245–1.3245)	0.8914 (0.4388–1.5669)	0.7620 (0.4170–1.1996)	0.502
(mIU·L^−1^)				
Growth hormone (μg·L^−1^)	0.356 (0.161–0.738)	0.349 (0.150–0.816)	0.377 (0.169–0.732)	0.802
Cortisol day 1				
Plasma cortisol (μg·dL^−1^)	13.89 (9.90–19.41)	12.82 (9.22–18.14)	15.66 (13.17–22.42)	0.039
24-h urinary free cortisol (μg·24 h^−1^)	548.10 (340.73–994.25)	527.73 (348.65–634.31)	683.13 (324.66–1,527.96)	0.141

*Data are presented as n (%) for categorical variables and as medians (interquartile ranges) for continuous variables. The bold values indicate the value of p < 0.05*.

*GCS, Glasgow Coma Scale; NIHSS, National Institutes of Health Stroke Scale; BMI, Body mass index; HR, Heart rate; SBP, Systolic blood pressure; DBP, Diastolic blood pressure; HbA1c, Hemoglobin A1c; LDL, Low-density lipoprotein; INR, International normalized ratio; CRP, C-reactive protein*.

### Comparison of hormone levels between different prognosis and severity groups

**Figure 2:**
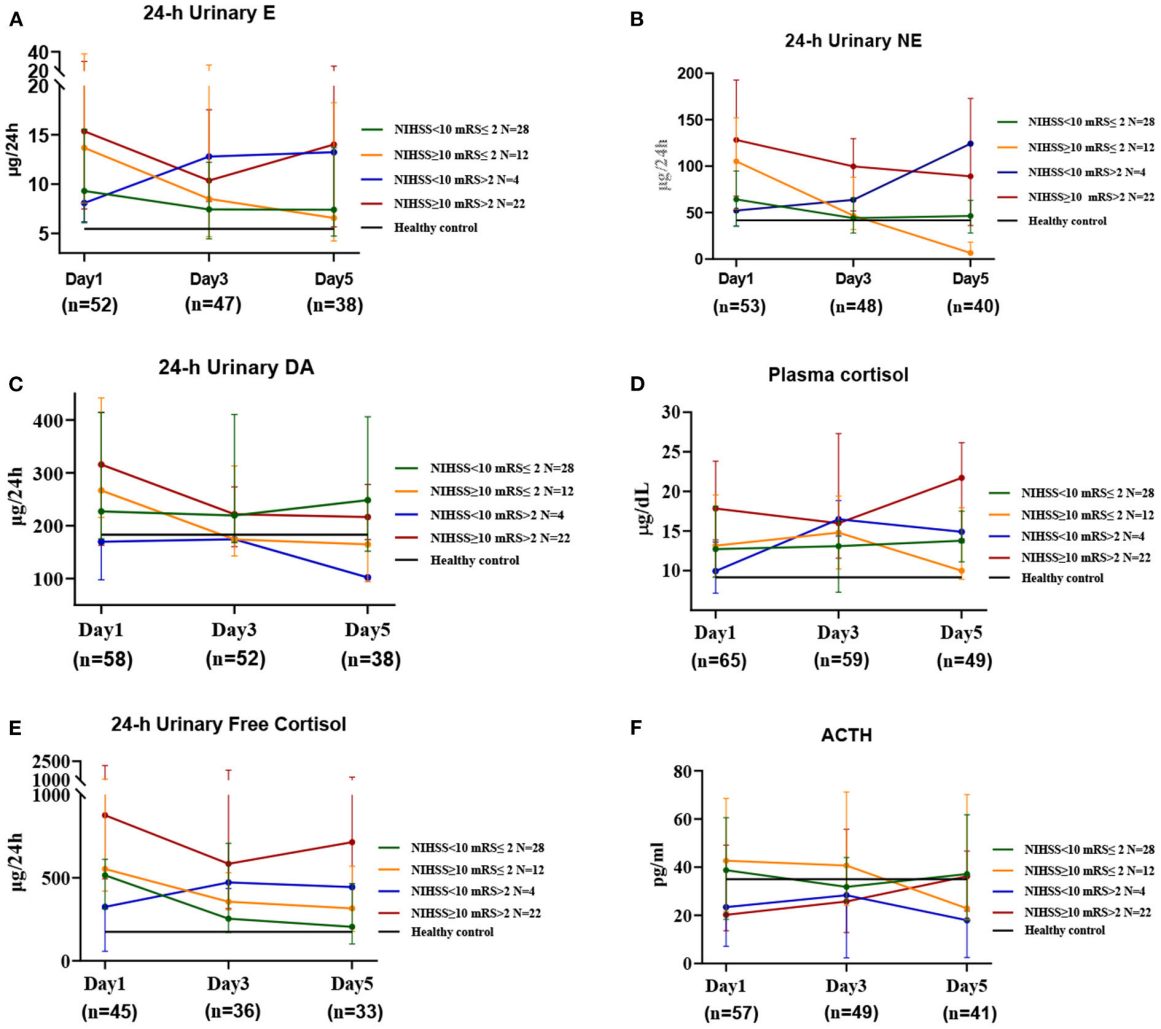
The changes in HPA axis hormones and catecholamines in stroke patients at three time periods. This includes the first day (Day 1), the third day (Day 3), and the fifth day after admission (Day 5). Median values for the 3 time periods show good and poor prognoses for patients with mild and severe disease at admission, respectively. Healthy controls show relative mean values, *n* = 237, age = 61.0 (± 9.5) years; *N*' refers to the number of cases in different prognosis and severity groups; *n*' refers to the number of specimens in different time periods.

Changes in hormone levels after stroke onset differed between the different prognosis and severity groups (Figure 2). On day 1 after the onset of stroke, regardless of prognosis or severity, patients showed increased levels of E, NE, F, and 24-h UFC, and over the next 5 days, E, NE, F, and 24-h UFC levels tended to decrease in patients with a good prognosis, while the levels tended to increase gradually or remained high in those with a poor prognosis. The levels of these four hormones were proportional to the severity (Supplementary Figure S3), with patients with severe disease and poor prognosis having the highest levels and those with mild disease and good prognosis having lower levels. The most significant decrease in NE levels was observed in patients with severe disease and good prognosis (2.5-fold on day 1, 1.1-fold on day 3 and 0.2-fold on day 5 compared to healthy controls, *P* = 0.012), while those with mild disease but poor prognosis showed an increasing trend in NE levels (1.2-fold on day 1, 1.5-fold on day 3 and 2.9-fold on day 5 compared to healthy controls). On day 1 after onset, DA levels were higher in the severe group than in the control group and then declined gradually to approach control levels by day 5. In contrast, DA levels in the mild group were comparable to those in the control group on days 1, 3, and 5. On day 1 after onset, ACTH levels were significantly lower in patients with a poor prognosis (0.6-fold) and higher in those with a good prognosis (1.2-fold) compared to the healthy controls and then gradually increased or decreased, approaching healthy control levels on day 5. Patients with a poor prognosis showed a gradual increase in ACTH levels after onset, while those with a good prognosis showed a gradual decrease. FSH and TSH levels were lower, and GH levels were higher in stroke patients than in healthy controls; FSH levels were higher in patients with a poor prognosis on day 1 than in those with a good prognosis, although TSH and GH levels were not significantly different. LH and PRL levels did not differ significantly between stroke and healthy control populations, regardless of prognosis or severity (Supplementary Figures S3, S4).

### Creation of receiver operating characteristic curves

**Figure 3:**
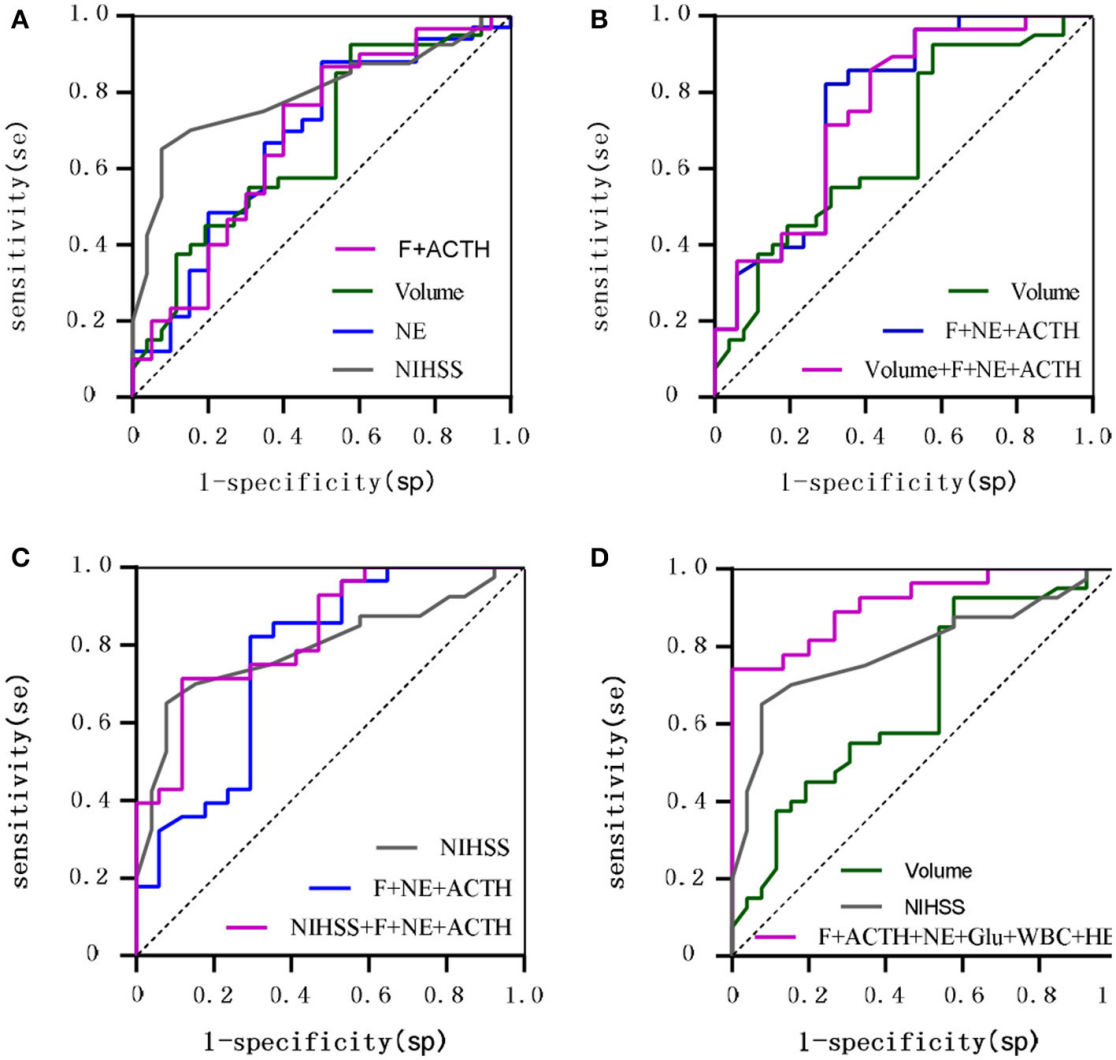
ROC curves comparing the value of hormones vs. traditional clinical variables for 90-day prognostic assessment. **(A)** Shows F combined with ACTH, NE, Lesion volume, NIHSS; **(B)** Shows F combined with NE and ACTH compared with Lesion volume; **(C)** Shows F combined with NE and ACTH compared with NIHSS; **(D)** Shows F combined with ACTH and NE and Glu and WBC and HB compared with Lesion volume, NIHSS.

To further investigate the value of biomarkers in assessing stroke prognosis, we plotted ROC curves by combining biomarkers measured on day 1 after onset with clinical variables (Supplementary Figure S5). The AUC values for F, NE, and ACTH were found to be similar to WBC, age and lesion volume and lower than the NIHSS score, the GCS score, Glu, and HB. After adjustment for age, sex and the NIHSS score, *F* > 13.6 μg/dL and ACTH > 22.02 pg/mL were found to be risk factors for poor stroke prognosis (*P* < 0.05), and after adjustment for age, sex and the GCS score, NE > 123.5 μg/24 h was identified as a risk factor for poor stroke prognosis (*P* < 0.05). The AUC for the combination of F and ACTH to predict poor stroke prognosis was comparable to that of lesion volume (0.682 vs. 0.682) and smaller than that of the NIHSS score (0.682 vs. 0.768), but the difference was not statistically significant (*P* = 0.367). The AUC (0.677 vs. 0.768) for NE in predicting poor stroke prognosis was comparable to that of lesion volume (0.677 vs. 0.680) and smaller than that of the NIHSS score, but the difference was not statistically significant (*P* = 0.260). The AUC (0.754 vs. 0.682 *P* = 0.359) and sensitivity (58.8% vs. 42.3%) were higher for the combination of F, ACTH, NE, and lesion volume in predicting poor prognosis of stroke than for lesion volume alone. The AUC (0.832 vs. 0.768 *P* = 0.190) and specificity (71.4% vs. 65.0%) were higher for the combination of F, ACTH, NE, and the NIHSS score than for the NIHSS score alone. The accuracy of the combination of F, ACTH, NE, WBC, Glu, and HB in predicting poor prognosis of stroke was significantly higher than that of lesion volume (AUC: 0.931 vs. 0.694 *P* = 0.019) and the NIHSS score (AUC: 0.931 vs. 0.746 *P* = 0.034) (Figure 3).

### The correlation of hormones with clinical variables

**Figure 4:**
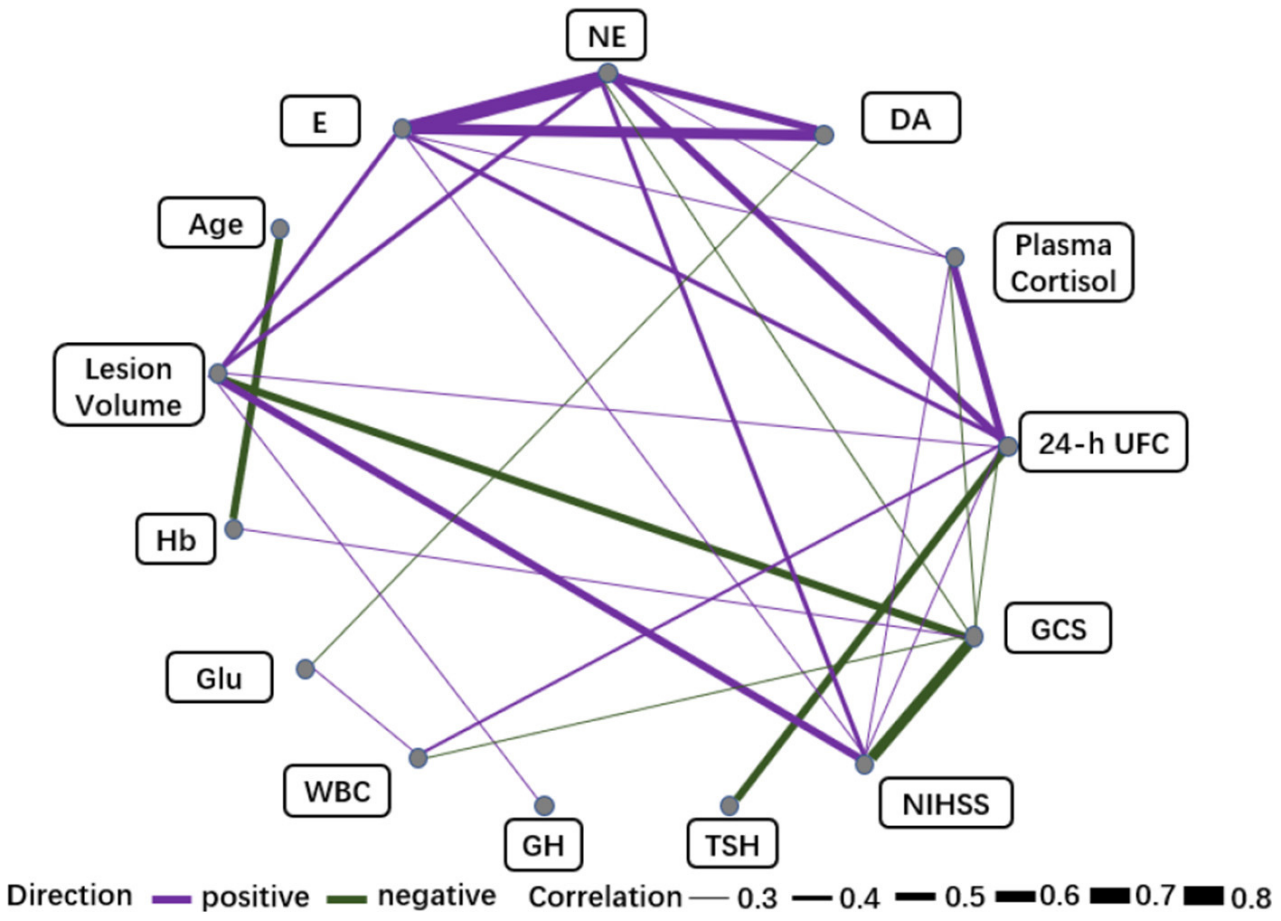
Correlation networks for biomarkers (Day 1) and clinical variables. The width of the edge showing stronger or weaker interactions is proportional to the absolute value of biomarker-biomarker correlation (|r|). Edges are shown only when |r| > 0.3. A purple edge indicates a positive correlation, and a green edge indicates a negative correlation.

We observed varying degrees of correlation of E and NE with F and 24-h UFC on day 1 post-stroke onset, with NE correlating most strongly with 24-h UFC (*r* = 0.54), 24-h UFC correlating negatively with TSH and PRL (*r* = −0.56, *r* = −0.43), and no significant correlation between catecholamines and pituitary hormones. E and NE were moderately positively correlated with lesion volume (*r* = 0.47, *r* = 0.40), and DA was weakly negatively correlated with Glu (*r* = −0.37). E, NE, F, and 24-h UFC were all correlated with the GCS and NIHSS scores to varying degrees, with the strongest correlation being between NE and the NIHSS score (*r* = 0.45). In addition, 24-h UFC was positively correlated with lesion volume and WBC (*r* = 0.36, *r* = 0.40) (Supplementary Figure S5), and NE and UFC were the nodes with a high concentration of 2 connecting lines (7 edges, 9 edges) among the biomarkers (Figure 4, see Supplementary Figure S6 for full information).

## Discussion

In the present study, we simultaneously assessed the dynamics of steroid hormones, catecholamine hormones and gonadotropins at the onset of stroke and their accuracy in predicting functional prognosis and mortality for acute ischemic stroke patients within 90 days of onset by mass spectrometry analysis of targeted metabolomics. Our main finding is that, in agreement with most published results, cortisol is an independent prognostic marker of functional prognosis and mortality in ischemic stroke patients (11). We demonstrated that cortisol levels increased with imaging lesion size and neurological deficits (assessed by the NIHSS), reflecting stroke severity. In contrast, TSH and GH, although also dynamically altered at stroke onset, showed limited variation, possibly because current measures are not sensitive enough to provide meaningful information on prognosis. It is very interesting to note the synergy between HPA hormones, catecholamine hormones and conventional test markers that can be used as an adjunct to imaging and the NIHSS score to assess clinical prognosis and provide important additional predictive information. For example, the accuracy of the combination of F and ACTH in predicting poor prognosis of stroke is comparable to that of lesion volume and second only to the NIHSS score, and the addition of F and ACTH to lesion volume and the NIHSS score increases the accuracy of lesion volume and the NIHSS score in predicting poor prognosis of stroke. Urine tests for catecholamines are also of some value; for example, the 24-h urinary NE level is a better assessor of severity and prognosis within 5 days of stroke onset and has shown similar accuracy to lesion volume in assessing poor stroke prognosis. If lesion volume and the NIHSS score were combined with F, ACTH, and NE markers, they would show higher sensitivity than lesion volume alone and higher specificity than the NIHSS score alone, thus improving the accuracy of prediction.

In addition, our study compared the above neuroendocrine metabolites in urine and plasma and found that 24-h UFC and F levels were significantly higher in stroke patients than in healthy subjects within 5 days of stroke onset, with 24-h UFC and F levels significantly higher in the poor prognosis group than in the good prognosis group and in the high severity group than in the low severity group. Within 5 days of stroke onset, 24-h UFC showed a more pronounced and stable trend than F. Moreover, 24-h UFC showed a similar value to F in assessing prognosis and severity. This is of great value in assessing the trend of disease progression following stroke onset and treatment and in guiding clinical management.

Quantitative stroke neuropathology with substantial quantification of the rate of neural circuit loss in acute ischemic stroke assessed by imaging tests has made possible a more objective and quantitative assessment of brain neuronal loss per unit time in acute brain injury to understand stroke severity and prognosis (21, 22). This study also yielded consistent results, finding that among the indicators tested, E, NE, 24-h UFC, and GH had the best correlation with MRI or CT brain tissue damage volume assessment at stroke onset for prognosis and disease severity, with E correlating most strongly with lesion volume (*r* = 0.468 *P* = 0.000), NE (*r* = 0.403 *P* = 0.003), *F* (*r* = 0.270 *P* = 0.035), 24-h UFC (*r* = 0.364 *P* = 0.014), and GH (*r* = 0.357 *P* = 0.011). More importantly, our results also used imaging test results as a control parameter for prognostic evaluation of stroke and found that some central endocrine metabolites, such as NE, had an assessment value similar to that of CT or MRI results to assist in the assessment of not only neuroimaging effects but also the dynamic changes in NE and other biomarkers in the acute phase of stroke. A temporal correlation was found for the assessment of stroke progression, treatment effects and prognosis, suggesting that the measurement of these indicators in blood and urine is a more effective adjunct to assessment tools for heterogeneous samples, with its lower cost, easy quantification methods, repeatability in a short period of time and reliable prediction of outcomes. In addition, CT or MRI combined with the measurement of central endocrine metabolites has a more objective and accurate value in improving the prognostic assessment of stroke.

There is growing clinical and experimental evidence of a causal relationship between brain injury and cardiac dysfunction. Most poststroke deaths are attributed to neurological injury, with cardiovascular complications being the second leading cause of death after stroke. Potential mechanisms of brain–heart interactions after stroke, such as the HPA axis, catecholamine surge, and sympathetic and parasympathetic regulation, are relevant, and there are clinical studies directly confirming that supplementation of these hormones to stroke patients has a beneficial effect on stroke. However, few studies have examined urinary levels of E, NE, and DA in the acute and subacute phases following stroke onset. Our study found that urinary E, NE, and DA levels rose significantly on the day of stroke onset and gradually decreased over the next 5 days but remained high. We therefore suggest that sympathetic arousal may persist in stroke patients for at least 5 days after stroke onset, thereby mediating cardiovascular complications leading to a poor prognosis. However, it is uncertain whether β-blockers and dopamine treatment can improve the outcome of stroke by altering sympathetic drive and increasing dopamine concentrations (23, 24).

The main cause of poor stroke prognosis is poststroke neuroendocrine disruption. Serum cortisol levels increase in proportion to the degree of stress and correlate with stroke severity. The reasons for the poor prognosis of stroke patients with high cortisol levels are related to the following: (1) Excess cortisol has been known to exacerbate ischemic neuronal damage, especially in the hippocampus. (2) Patients with stroke and high cortisol levels are more likely to experience cardiac events (e.g., arrhythmias or myogenic fibrous degeneration), resulting in higher mortality (8). This result of our study is consistent with the results of other studies. Additionally, our study demonstrated the same changes in urinary cortisol levels.

Although in some prospective studies, stroke patients had significantly higher serum GH levels on admission than their normal counterparts, showing a significant correlation with 30-day mortality and 90-day functional recovery, GH levels on admission were considered to be an independent predictor of patient mortality (10). Our study did not show a statistically significant difference, although we also observed a significant increase in GH levels after stroke and a stepwise decrease in GH levels after treatment in patients with prognosis. This may be related to our small sample size and overly specific subgroups, but our results suggest that GH is slightly less sensitive than catecholamines and glucocorticoids in predicting the severity and prognosis of stroke. This may be related to the fact that GH levels are elevated not only in stroke patients but also in critically ill adult patients caused by severe infections of the central nervous system and sepsis, so GH reflects disease severity and patient stress status (25).

Similarly, in our study, TSH was less sensitive than catecholamines in predicting stroke, and although TSH levels decreased significantly after stroke onset and showed a gradual increase within 5 days of stroke onset, TSH did not show value in predicting poor prognosis or assessing severity.

In summary, we suggest that the intensity of the neuroendocrine response may be a potential prognostic marker for brain injury (26), and that these endocrine-related biomarkers combined with imaging and NIHSS scores are valuable in assessing the prognosis of stroke.

### Limitations

Stroke is highly heterogeneous, and this study did not include a multifactorial analysis to demonstrate that these biomarkers are independent prognostic indicators due to sample size limitations. To minimize the effect of interference of confounding factors on the results, we set strict inclusion criteria to screen all patients for any comorbidities or any medication use that could affect CA levels, which limited the number of eligible patients in this study and could have led to selection bias. We did not assess patients' basal levels of TSH, GH, and CAs prior to the onset of the disease, such as the presence of subclinical hypothyroidism in patients, which would have affected the results of the study. The secretion of anterior pituitary hormones is rhythmic and is influenced by external environmental factors, such as season, light and darkness, and the sleep-wake cycle [e.g., GH secretion is pulsatile, and TSH levels vary by up to approximately 50% on average from day to day (27)] and by sex and age (28). These factors are inevitable, although there was no difference in the sex ratio between the stroke and control groups and all women were menopausal; we also had a dedicated area with a quiet and normal environment and made efforts to maintain a normal sleep-wake cycle for patients and collect blood and urine specimens at a uniform time.

## Conclusion

Although the exact mechanism has not been elucidated, the present study suggests that stroke causes significant temporal and dynamic changes in the HPA axis pathway and sympathetic nervous system (SNS) hormones and that plasma F, ACTH, and urinary NE levels can be used to assess stroke severity and prognosis. These findings need to be confirmed by cohort studies with larger samples and longer follow-up, and future trials are necessary to demonstrate viable options for improving stroke prognosis through treatments that improve the endocrine response.

## Data Availability Statement

The original contributions presented in the study are included in the article/Supplementary material, further inquiries can be directed to the corresponding author/s.

## Ethics Statement

The studies involving human participants were reviewed and approved by ethical approval for the study was obtained from Ethics Committee of Ruijin Hospital North, Shanghai Jiao Tong University School of Medicine (2019-002-2). The patients/participants provided their written informed consent to participate in this study.

## Author contributions

Y-ML and JYe contributed to conception and design of the study. LH, YC, H-WS, LZ, and Z-BL organized the database. HW and X-SW performed the statistical analysis. X-GC and S-YS wrote the first draft of the manuscript. JYu and Y-JZ wrote sections of the manuscript. All authors contributed to manuscript revision, read, and approved the submitted version.

## Funding

Work in the JYe/Y-ML laboratories was supported by the National Natural Science Foundation of China (Grant Numbers 81971312, 91749126, 81911530241, 81871549, and 81671900), the Program of Shanghai Academic/Techonology Research Leader (Grant Number 19XD1422500), and the Shanghai Municipal Education Commission (Oriental Scholars Program, 2019).

## Conflict of interest

The authors declare that the research was conducted in the absence of any commercial or financial relationships that could be construed as a potential conflict of interest.

## Publisher's note

All claims expressed in this article are solely those of the authors and do not necessarily represent those of their affiliated organizations, or those of the publisher, the editors and the reviewers. Any product that may be evaluated in this article, or claim that may be made by its manufacturer, is not guaranteed or endorsed by the publisher.
