# Supplementary material for: Longitudinal changes in the hypothalamic–pituitary–adrenal axis and sympathetic nervous system are related to the prognosis of stroke
Source: Front Neurol. 2022 Jul 27;13:946593. doi: 10.3389/fneur.2022.946593 (PMC9364825; doi:10.3389/fneur.2022.946593)
Supplement: Supplementary file 1 [file Image_1.pdf]

Online Supplement

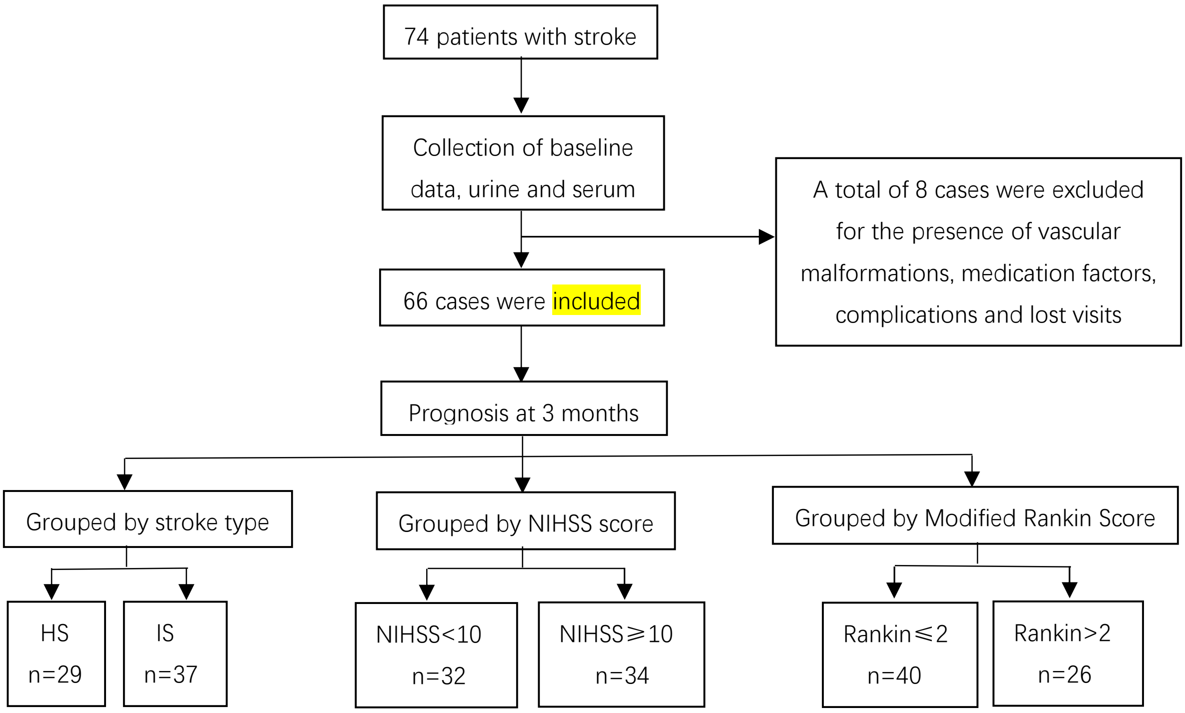

Supplementary Figure 1. Patient screening and grouping

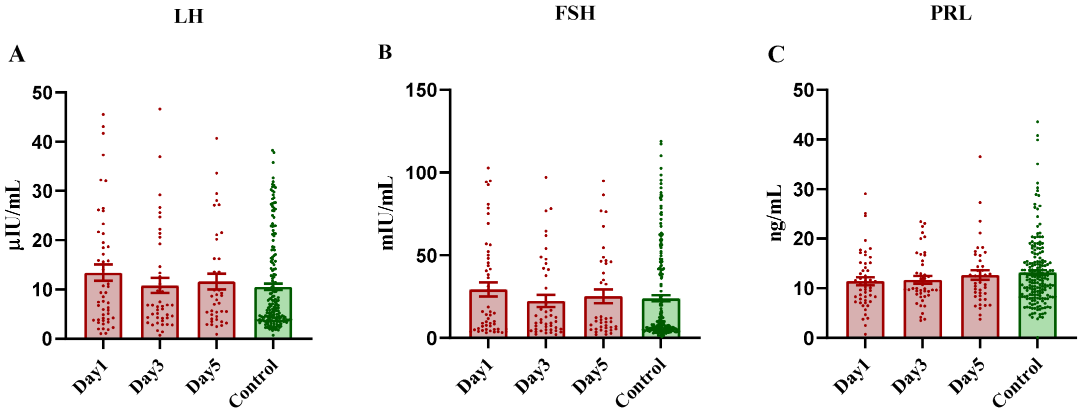

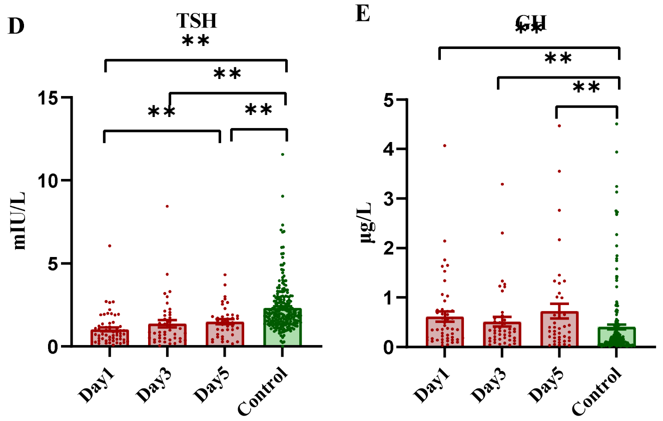

Supplementary Figure 2 Scatterplot of early levels of each hormone in stroke versus healthy control (median and interquartile range)

Note: * denotes *P*<0.05, ** denotes *P*<0.01.

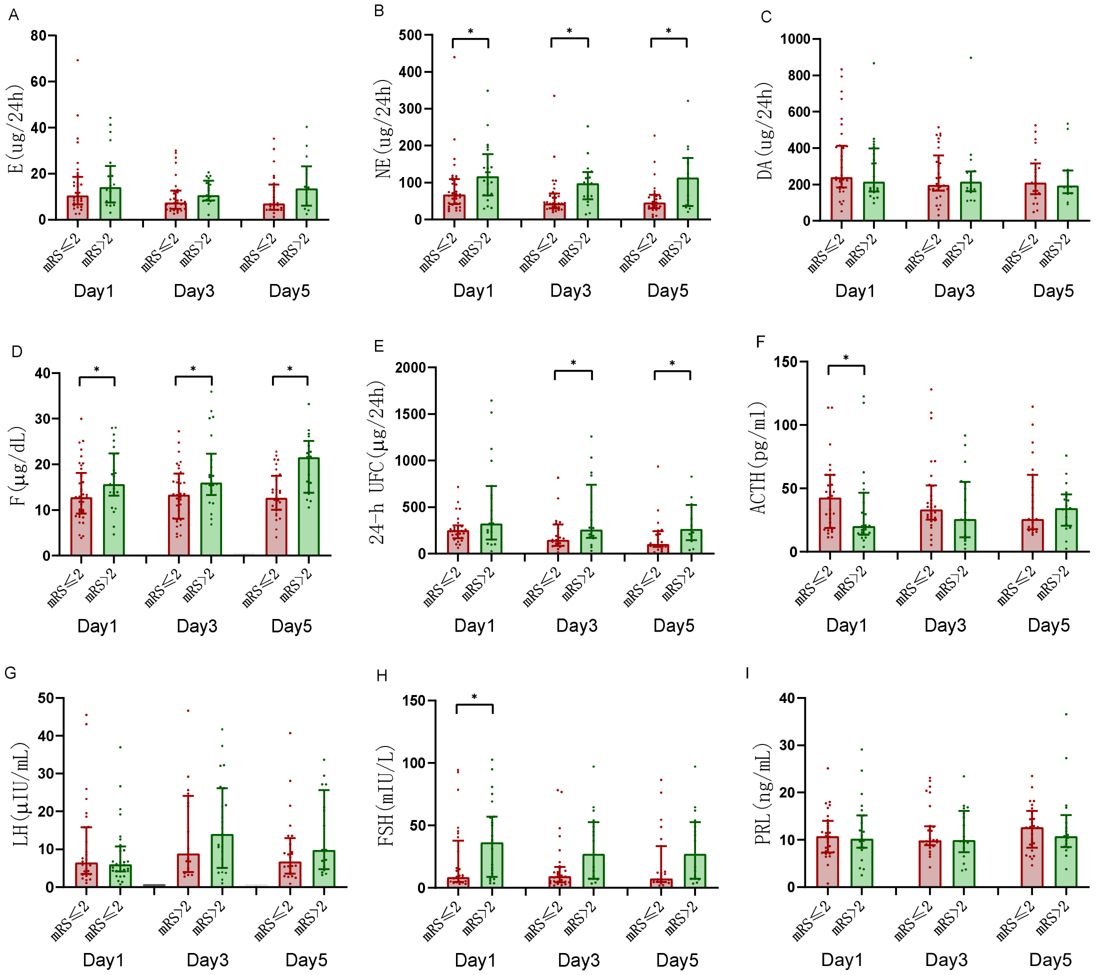

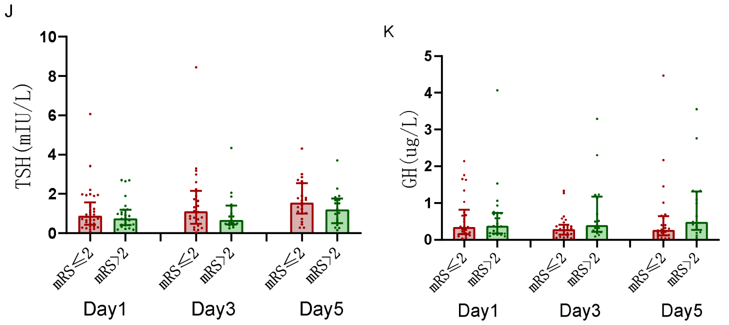

Supplementary Figure 3 Comparison of individual hormone levels in different prognostic groups

Note: * denotes P<0.05

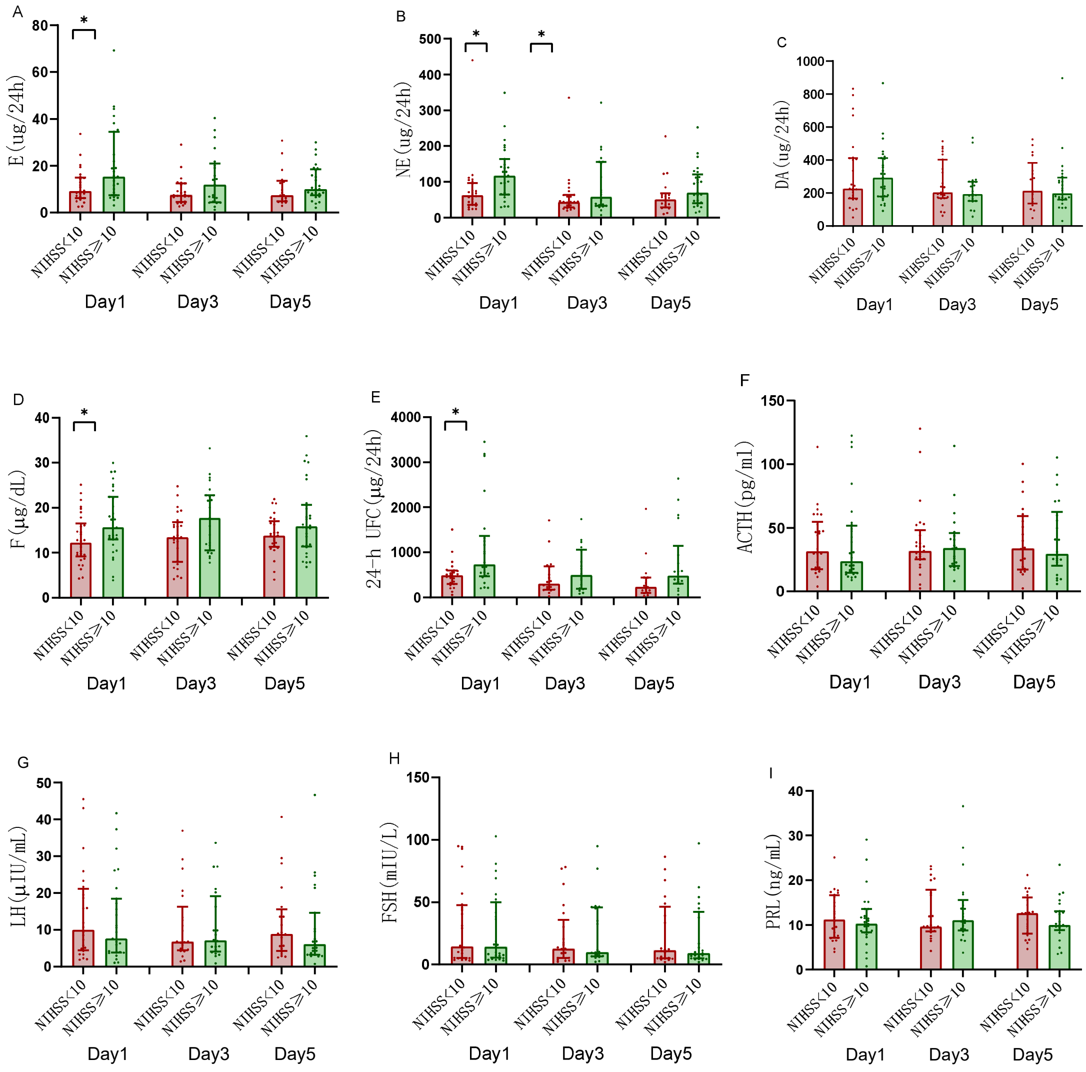

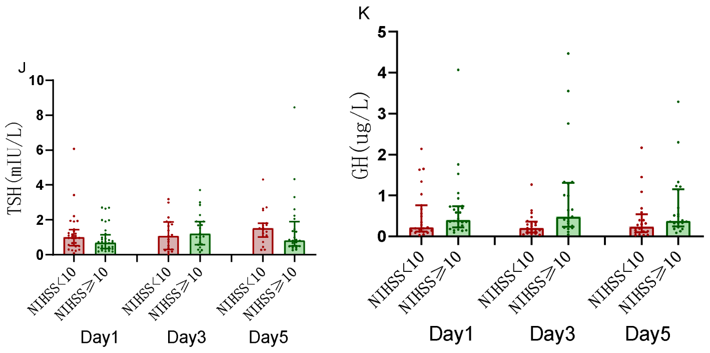

Supplementary Figure 4 Comparison of hormone levels at various time points in different severity groups.

* indicates a significant difference between groups（p<0.05）

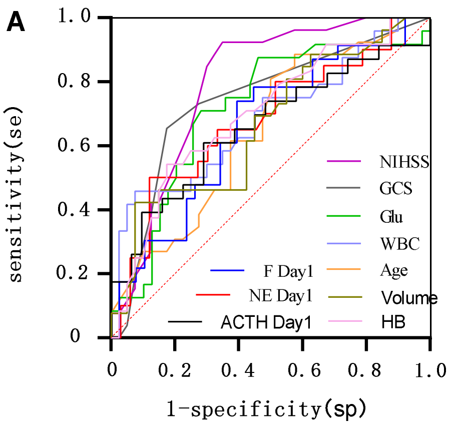

B

Variable	AUC	Cut off	*P*
NIHSS	0.7938	13	<0.001
GCS	0.7404	8	0.001
Glu	0.7147	7.7	0.004
WBC	0.6823	9.96	0.015
Age	0.6481	53	0.043
Volume	0.6635	23.75	0.026
HB	0.7000	128	0.008
F Day1	0.6590	13.6	0.039
NE Day1	0.6765	123.5	0.033
ACTH Day1	0.6592	22.02	0.047

Supplementary Figure 5 ROC curves for poor prognostic risk factors

Supplementary Table 1 Analysis of the correlation between hormones and hormones and between hormones and clinical variables

		NE1	DA	UFC	F	FSH	PRL	TSH	WBC	Glu	HB	V	Age	NIHSS	GCS
E	r	.780	.600	.447	.331							.468		.363	
	t	.000	.000	.003	.018							.000		.008	
NE1	r		.542	.538	.362							.403		.454	-.335
	t		.000	.000	.008							.003		.001	.014
DA	r									-.374		.260			
	t									.008		.060			
UFC	r				.560		-.432	-.556	.397			.364		.346	-.306
	t				.000		.005	.000	.008			.014		.020	.041
F	r								.290			.270		.354	-.305
	t								.026			.035		.005	.017
ACTH	r					-.293					.322				
	t					.037					.020				
LH	r					.851	.322				-.340		.525		
	t					.000	.021				.017		.000		
FSH	r						.311				-.463		.664		
	t						.026				.001		.000		
PRL	r												.281		
	t												.046		
TSH	r								-.273			-.250			
	t								.036			.052			
GH	r											.357			
	t											.011			
WBC	r									.362		.297		.250	-.315
	t									.004		.017		.046	.011

HB	r	-.504		.337
	t	.000		.007
V	r		.557	-.515
	t		.000	.000
NIHSS	r			-.606
	t			.000

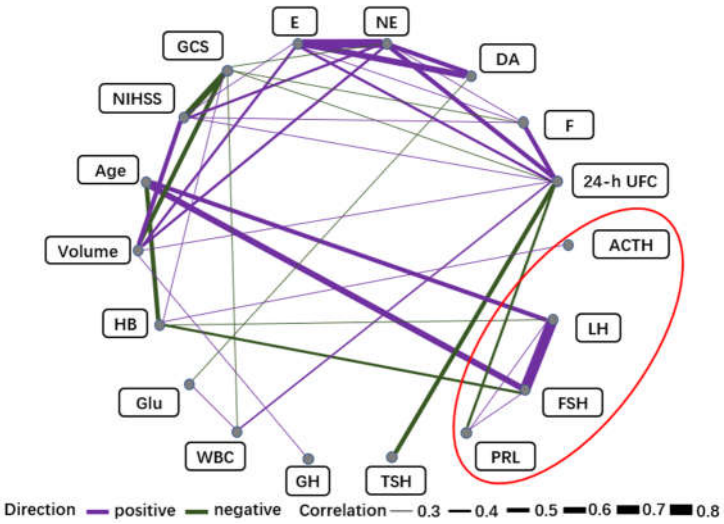

Supplementary Figure 6 Correlation analysis of all hormones with clinical variables

The red circle indicates the four markers added on the basis of Figure 4.
